# Supplementary material for: Metagenomic analysis reveals unexplored diversity of archaeal virome in the human gut
Source: Nat Commun. 2022 Dec 29;13:7978. doi: 10.1038/s41467-022-35735-y (PMC9800368; doi:10.1038/s41467-022-35735-y)
Supplement: Supplementary file 1 — Supplementary information [file 41467_2022_35735_MOESM1_ESM.pdf]

Title: Metagenomic analysis reveals unexplored diversity of archaeal virome in the human gut

Running title: Archaeal virome in the human gut

Ran Li[1,2,4], ran.li@siat.ac.cn

Yongming Wang[1,4], ym.wang2@siat.ac.cn

Han Hu[3], hanh@xbiom.com

Yan Tan[3], yant@xbiom.com

Yingfei Ma[1*], yingfei.ma@siat.ac.cn

[1]Shenzhen Key Laboratory of Synthetic Genomics, Guangdong Provincial Key Laboratory of Synthetic Genomics, CAS Key Laboratory of Quantitative Engineering Biology, Shenzhen Institute of Synthetic Biology, Shenzhen Institutes of Advanced Technology, Chinese Academy of Sciences, Shenzhen 518055, China

[2]University of Chinese Academy of Sciences, Beijing, 100049, China

[3]Xbiome, Scientific Research Building, Tsinghua High-Tech Park, Shenzhen, China

[4] These authors contribute equally.

*Correspondence to yingfei.ma@siat.ac.cn

Supplementary Information

Identification of archaeal genomic contigs from the metagenomes expands the archaeal diversity in the human gut

We identified the human-associated archaeal genome contigs from 12 human microbial metagenomic datasets consisting of 3,971 samples from rural and urban human populations across 13 countries (Supplementary Data 1, Supplementary Fig. 1), resulting in 17,830 archaeal genomic contigs from the human gut samples (Supplementary Fig. 2), but only 33 from the samples of other body sites (detailed in Methods, Supplementary Fig. 3). Thus, we focused on the archaea inhabiting the human gut. These contigs were taxonomically assigned based on the taxonomic information of the encoding proteins using the GTDB taxonomy system [1] (detailed in Methods). The result revealed a remarkably high taxonomic diversity of as-yet undescribed archaea in the human gut across 4 phyla including Methanobacteriota (72.76%), Thermophlasmatota (27.10%), Halobacteriota (0.10%) and Altarchaeota (0.03%), 8 families, 22 genera and 56 species (Supplementary Data 2). To further validate this result, these 17830 archaeal contigs were mapped to the 1,162 classified human gut archaeal genomes collected in UHGG (Unified Human Gastrointestinal Genome)[2] with BLASTn (E-value $\leqslant$ 10-5 and coverage $\geqslant$ 0.5, Supplementary Data 3a). The result showed that 15,732 contigs were matched to 833 gut archaeal genomes (Supplementary Data 3b, Supplementary Fig. 2b), while 2,098 (11.8%) did not yield a significant match in the 1,162 reference genomes. These 833 genomes were classified into 3 families (with 7 genera and 13 species) (Supplementary Fig. 2b). Most genomes were taxonomically affiliated with the genus Methanobrevibacter_A (735 genomes; 88.24%), in agreement with earlier reports[3]. Other genomes were affiliated to Candidatus Methanomethylophilaceae UBA71 (44; 5%), *Methanomethylophilus* (19; 2.3%), *Methanosphaera* (17; 2%), Methanomassiliicoccus_A (11; 1.3%) and Methanocorpusculum MX-02 (6; 0.7%). The discrepancy in the number of the archaeal taxa assigned by these two methods suggested that more novel archaea with extremely low abundance likely are present in the human gut.

Then, these 17,830 archaeal genomic contigs were de-replicated by clustering according to 95% average nucleotide identity (ANI) and only the contigs with length ≥ 3 kbp were kept. The longest contig within each cluster was chosen as the representative sequence, resulting in 2,948 nonredundant archaeal genomic contigs. We assessed the prevalence of these archaea in the human populations based on the number of metagenomic sequencing reads mapped to the representative sequences (Supplementary Fig. 2c). Due to raw reads of some HMP samples are not available, the total reads we used for the following analysis were derived from 1,904 metagenomic samples (Supplementary Data 4). As a result, a total of

1,770 (92.26%) samples had at least one read that was mapped to the archaeal contigs. It turned out that the most prevalent archaeal genera in the human gut were Methanobrevibacter_A (82.14%), followed by Candidatus Methanomethylophilaceae ISO4-G1 (74.32%), Candidatus Methanomethylophilaceae UBA71 (58.56%), *Methanomethylophilus* (28.2%) and *Methanosphaera* (20.27%), indicating the most common archaea in the human intestine.

Supplementary Figures

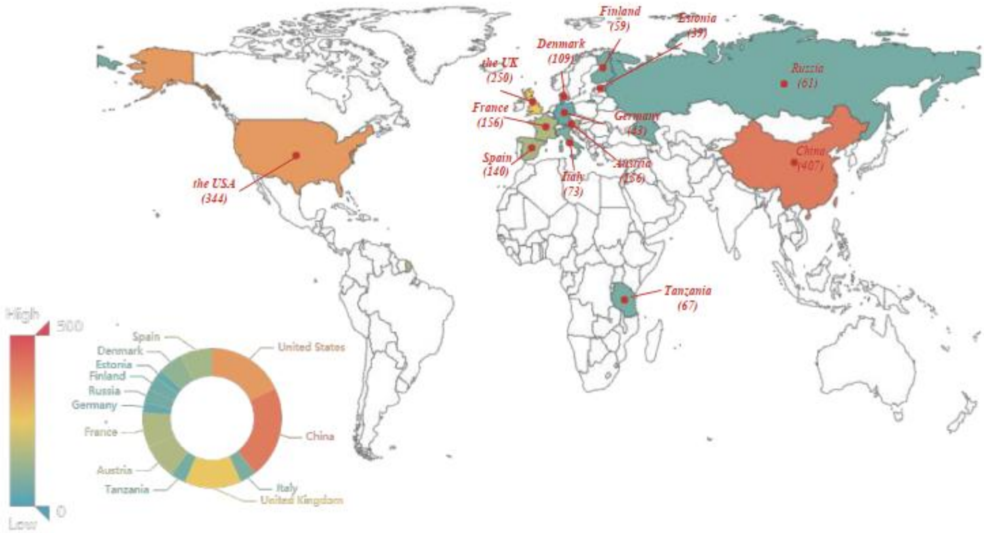

Supplementary Fig. 1 Overview of the metagenomic datasets selected in this study. Global heatmap of the world showing the number and distribution of studies per country was plotted using Python library matplotlib[4]. Source data are provided as a Source Data file.

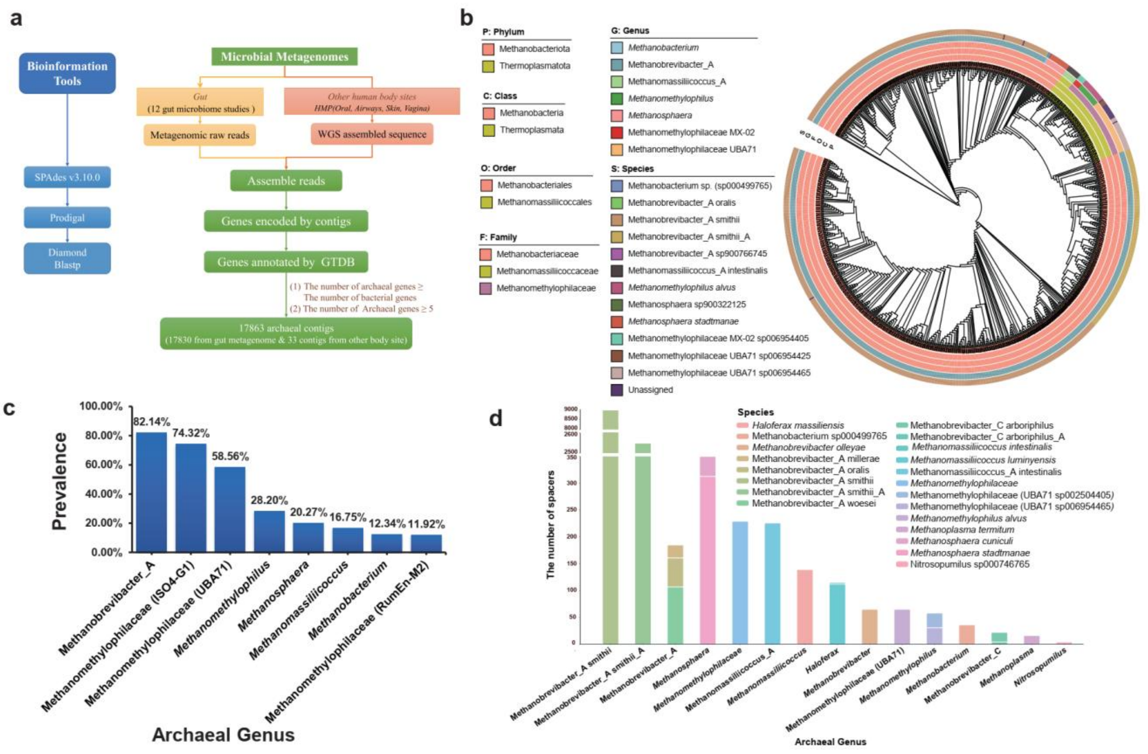

Supplementary Fig. 2 Identification of the human gut archaeal genomic contigs and recruitment of the CRISPR spacers. a, Workflow to identify archaeal genomic contigs from the assembled metagenomic sequencing data. b, Phylogenetic tree of the archaeal genomes mapped by the archaeal genomic contigs. This tree shows the diversity of the archaeal genomes recovered from the human gut. Branches of the tree are colored according to the archaeal taxa. c. Prevalence of the archaeal genera in the human population. d. The number of CRISPR spacers per archaeal host genus. The height of the bars displays the number of spacers identified from the identified archaeal contigs and the UHGG archaeal genomes belonging to the genus that corresponds to the horizontal axis. Different colors in the same bar represent different species belonging to the genus that corresponds to the horizontal axis. Source data are provided as a Source Data file.

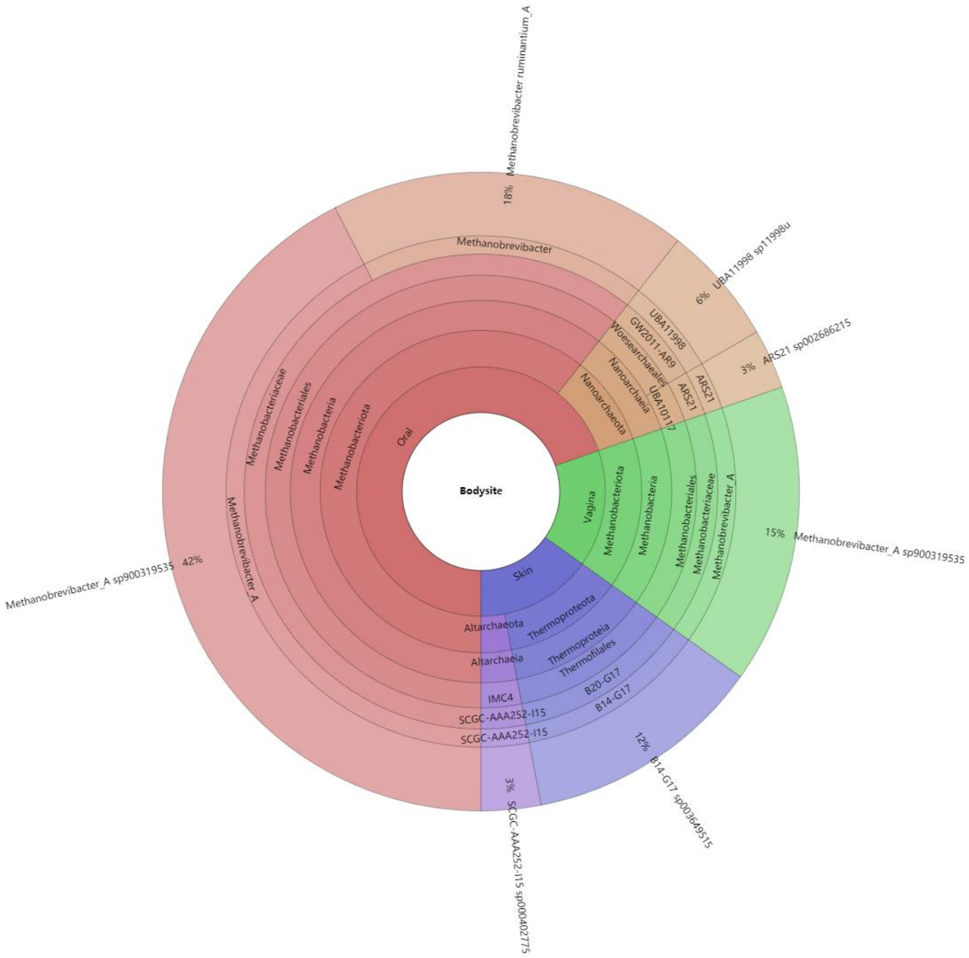

Supplementary Fig. 3 Concentric pie chart displayed by Krona showing the number of archaeal phyla (inner) and species (outer) detected from oral, skin, and vagina, respectively. Source data are provided as a Source Data file.

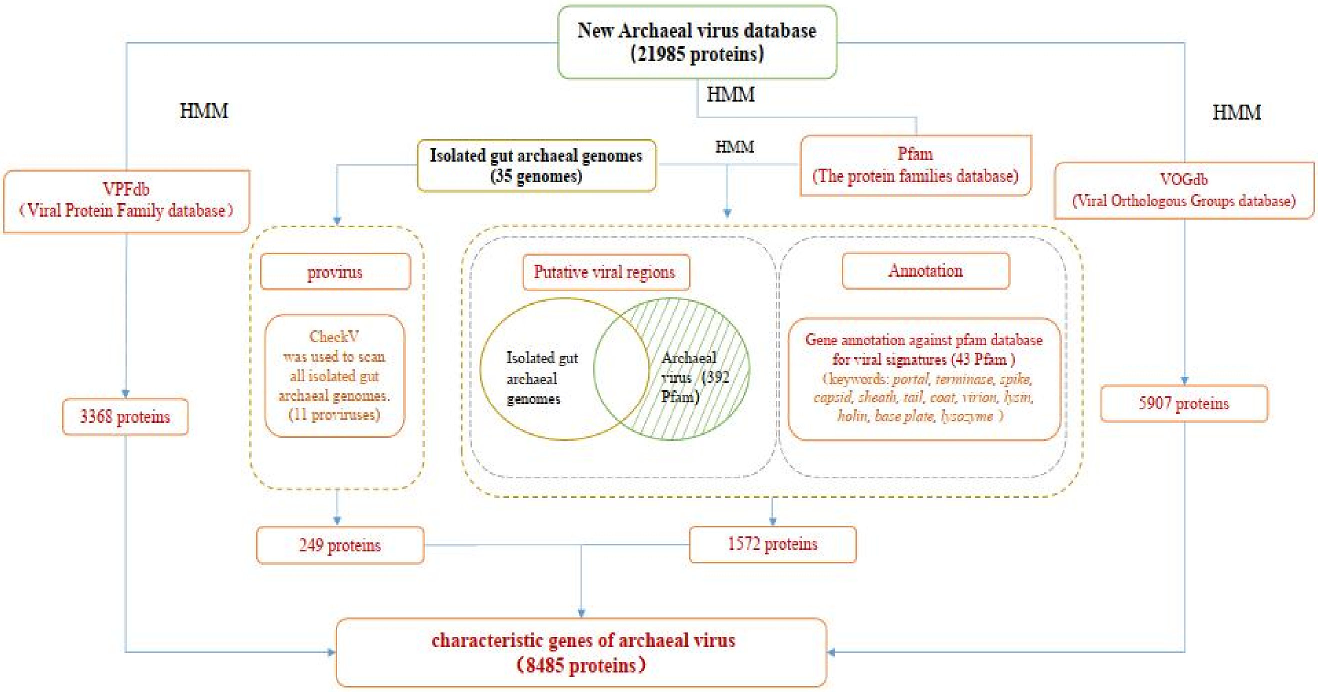

Supplementary Fig. 4 Workflow of archaeal viral hallmark gene identification. See the Methods for detailed information.

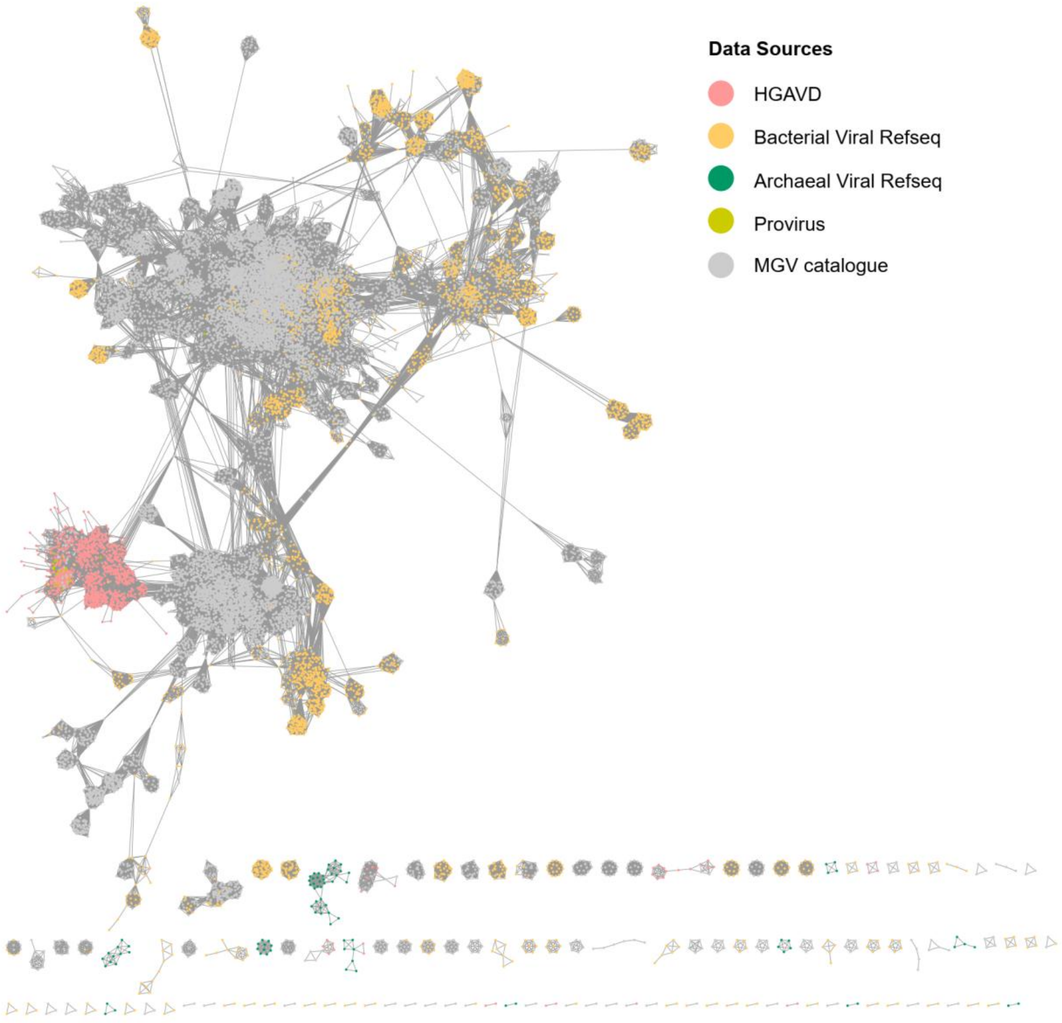

Supplementary Fig. 5 The whole protein-sharing network of the archaeal viruses in the human gut using vConTACT v2.0. The nodes and the connecting edges represent viral populations and their shared proteins, respectively. Nodes are depicted in the color representing viruses from the metagenomic samples (pink), Bacterial Viral Refseq (yellow), Archaeal Viral Refseq (blue), provirus identified from archaeal genomes (green), and viruses collected from the MGV catalog (gray). Source data are provided as a Source Data file.

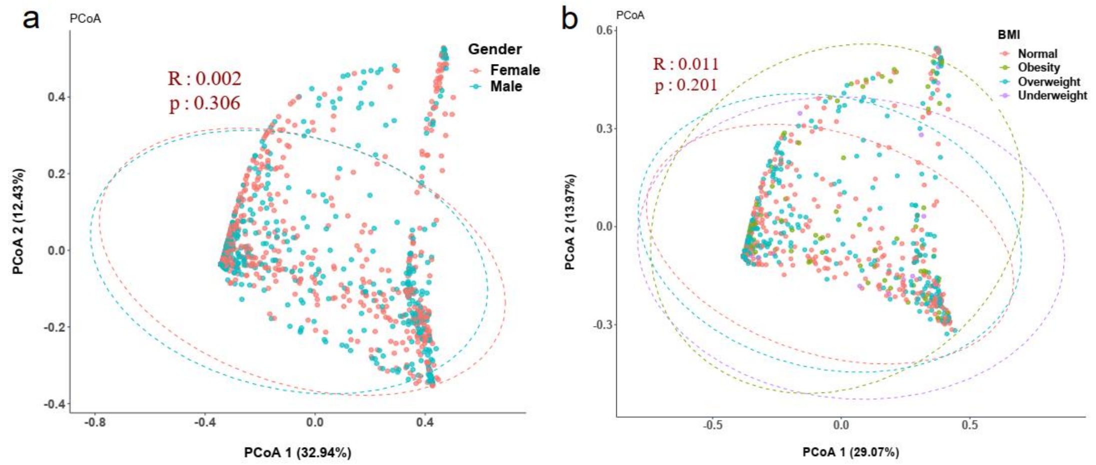

Supplementary Fig. 6 PCoA plot of inter-samples. Principal component analysis (PCoA) of Bray-Curtis distance matrix calculated from the viral abundance matrix. Each point is colored according to the gender and BMI of individuals. R and *p* values were obtained by a two-way analysis of similarities (ANOSIM). Source data are provided as a Source Data file.

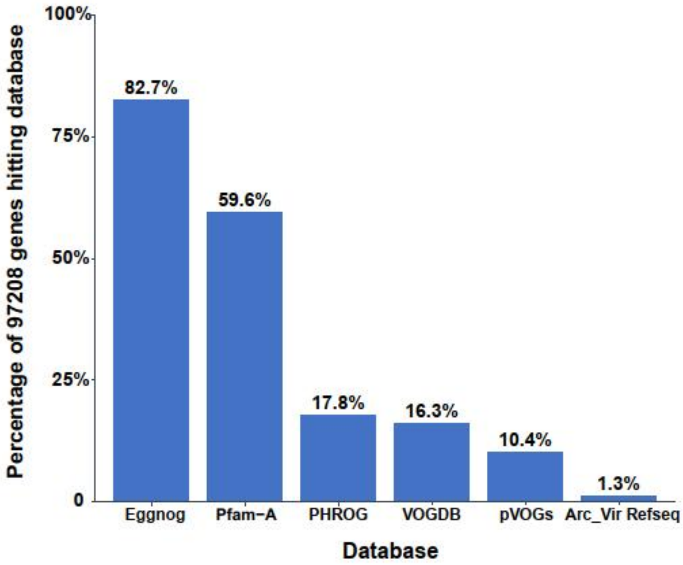

Supplementary Fig. 7 Protein-coding viral genes were identified for HGAVD and compared with profile HMMs five databases (Eggnog, Pfam-A, PHROG, VOGDB, pVOGs) and Archaeal Viral Refseq.

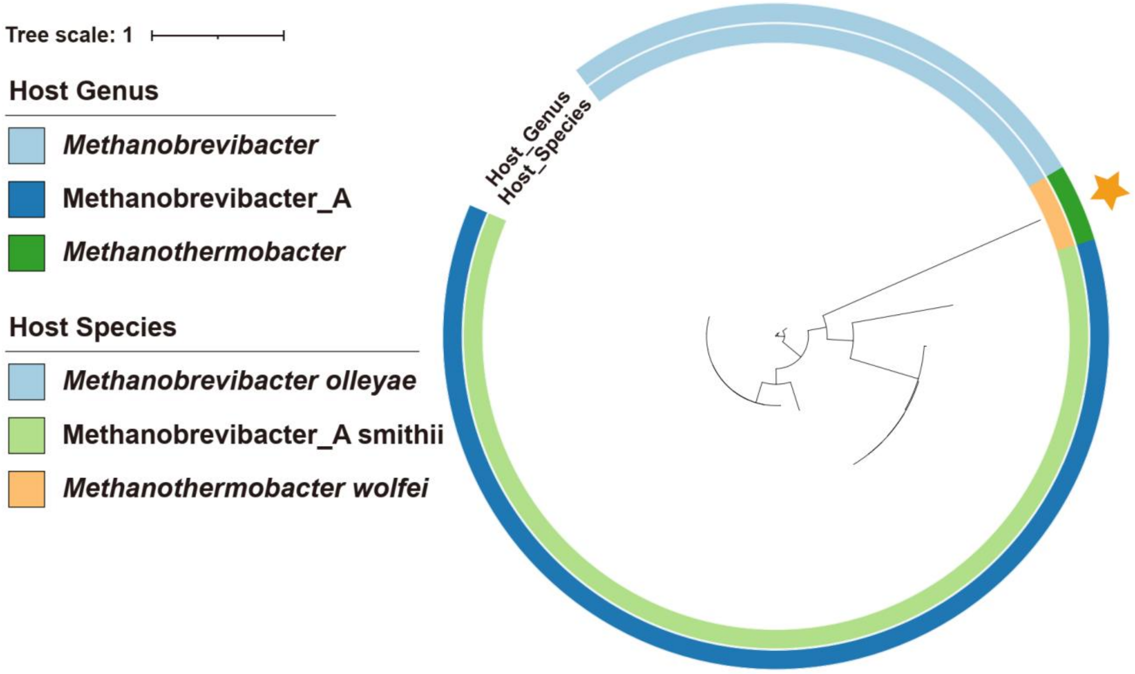

Supplementary Fig. 8 Maximum likelihood phylogenetic tree of PeiW proteins encoded by the complete genomes of the HGAVD viral species. The tree was constructed using the automatic optimal model selection. The star shows the prototype protein of PeiW (UniProtKB Q7LYX0).

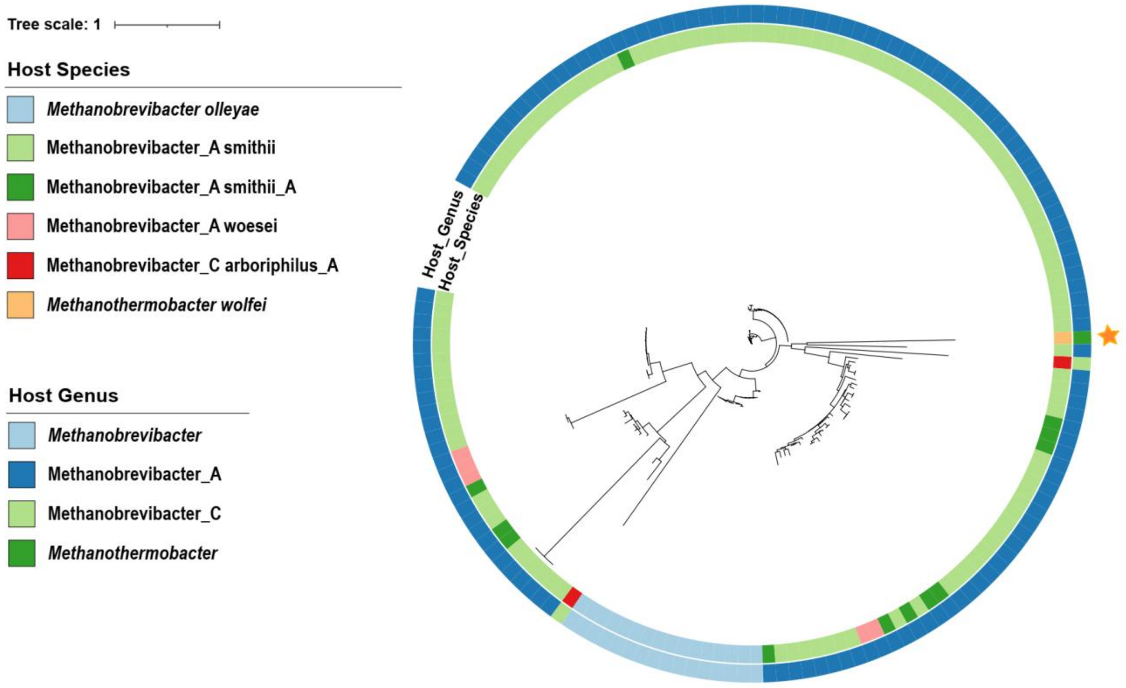

Supplementary Fig. 9 Maximum likelihood phylogenetic tree of PeiW proteins encoded by HGAVD viruses. The tree was constructed using the automatic optimal model selection. The star shows the prototype protein of PeiW (UniProtKB Q7LYX0).

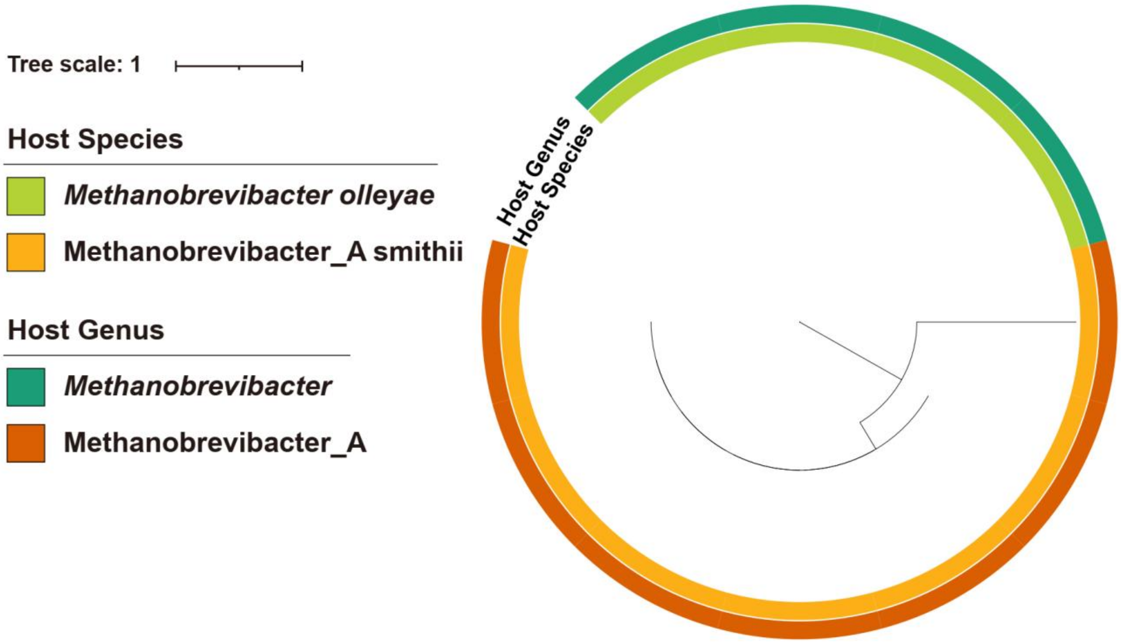

Supplementary Fig. 10 Maximum likelihood phylogenetic tree of MazE-antitoxin proteins encoded by the complete genomes of the HGAVD viral species.

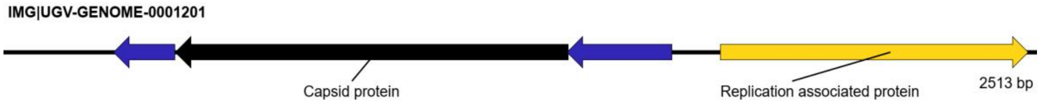

Supplementary Fig. 11 Genetic maps of the longest genome from the orders *Cremevirales* in HGAVD. The arrows depict the location and direction of predicted proteins on the viral genomes, and the filled colors indicate different gene functional categories, as depicted in the legend. The annotations were based on searches against the nr database, and only significant results (e-value < 1e-5) are shown.

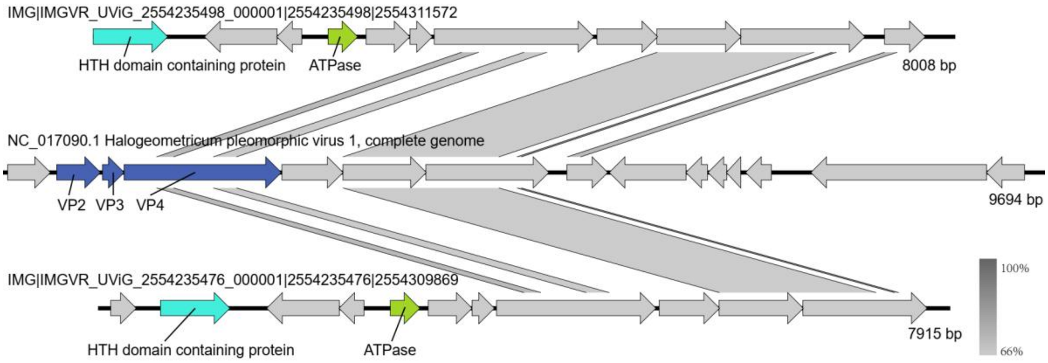

Supplementary Fig. 12 Schematic genomic alignment of the two representative genome from the orders Haloruvirales in HGAVD and the Halogeometricum pleomorphic virus 1 HGPV-1 (GenBank accession JN882267) as linear representation. HGPV-1 genome is used as a reference. The arrows depict the location and direction of predicted proteins on the viral genomes, and the filled colors indicate different gene functional categories, as depicted in the legend. The annotations were based on searches against the nr database, and only significant results (e-value < 1e-5) are shown.

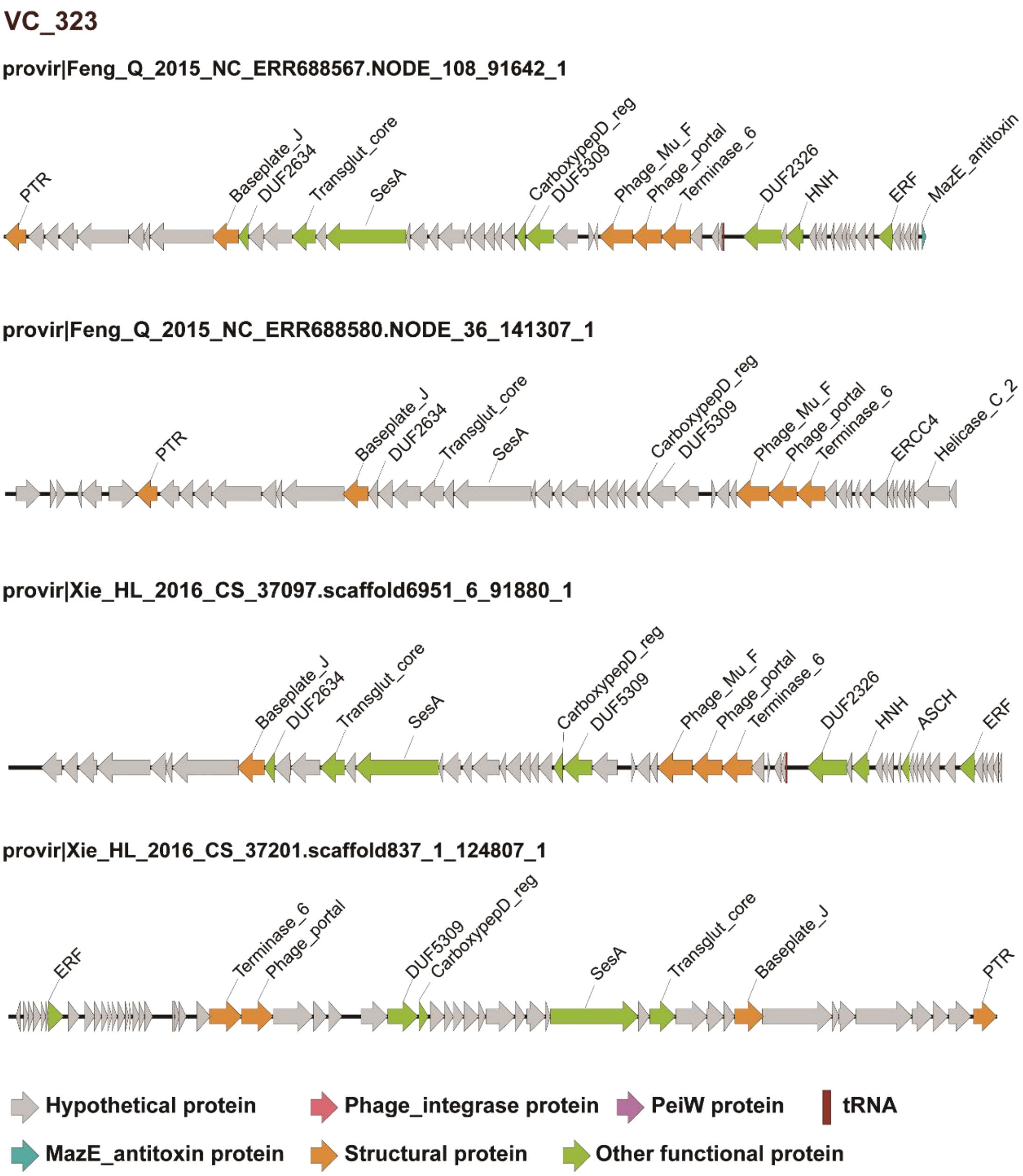
VC_323
provir|Feng_Q_2015_NC_ERR688567.NODE_108_91642_1
PTR
Baseplate_J
DUF2634
Transglut_core
SesA
CarboxypepD_reg
DUF5309
Phage_Mu_F
Phage_portal
Terminase_6
DUF2326
HNH
ERF
MazE_antitoxin
provir|Feng_Q_2015_NC_ERR688580.NODE_36_141307_1
PTR
Baseplate_J
DUF2634
Transglut_core
SesA
CarboxypepD_reg
DUF5309
Phage_Mu_F
Phage_portal
Terminase_6
ERCC4
Helicase_C_2
provir|Xie_HL_2016_CS_37097.scaffold6951_6_91880_1
Baseplate_J
DUF2634
Transglut_core
SesA
CarboxypepD_reg
DUF5309
Phage_Mu_F
Phage_portal
Terminase_6
DUF2326
HNH
ASCH
ERF
provir|Xie_HL_2016_CS_37201.scaffold837_1_124807_1
ERF
Terminase_6
Phage_portal
DUF5309
CarboxypepD_reg
SesA
Transglut_core
Baseplate_J
PTR
Hypothetical protein
Phage_integrase protein
PeiW protein
tRNA
MazE_antitoxin protein
Structural protein
Other functional protein

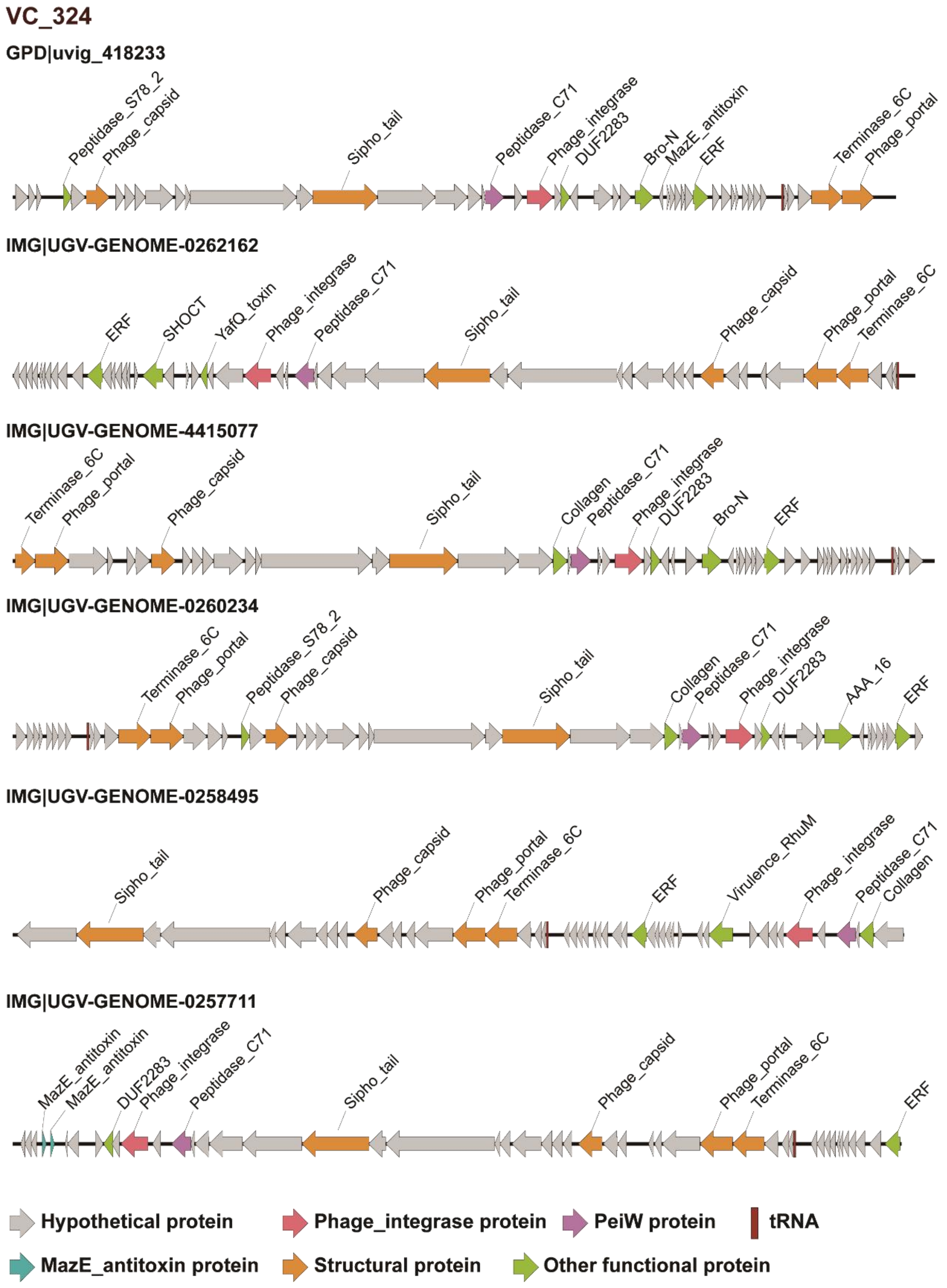
VC_324
GPD|uvig_418233
Peptidase_S78_2
Phage_capsid
Sipho_tail
Peptidase_C71
Phage_integrase
DUF2283
Bro-N
MazE_antitoxin
ERF
Terminase_6C
Phage_portal
IMG|UGV-GENOME-0262162
ERF
SHOCT
YafQ_toxin
Phage_integrase
Peptidase_C71
Sipho_tail
Phage_capsid
Phage_portal
Terminase_6C
IMG|UGV-GENOME-4415077
Terminase_6C
Phage_portal
Phage_capsid
Sipho_tail
Collagen
Peptidase_C71
Phage_integrase
DUF2283
Bro-N
ERF
IMG|UGV-GENOME-0260234
Terminase_6C
Phage_portal
Peptidase_S78_2
Phage_capsid
Sipho_tail
Collagen
Peptidase_C71
Phage_integrase
DUF2283
AAA_16
ERF
IMG|UGV-GENOME-0258495
Sipho_tail
Phage_capsid
Phage_portal
Terminase_6C
ERF
Virulence_RhuM
Phage_integrase
Peptidase_C71
Collagen
IMG|UGV-GENOME-0257711
MazE_antitoxin
MazE_antitoxin
DUF2283
Phage_integrase
Peptidase_C71
Sipho_tail
Phage_capsid
Phage_portal
Terminase_6C
ERF
Hypothetical protein
Phage_integrase protein
PeiW protein
tRNA
MazE_antitoxin protein
Structural protein
Other functional protein

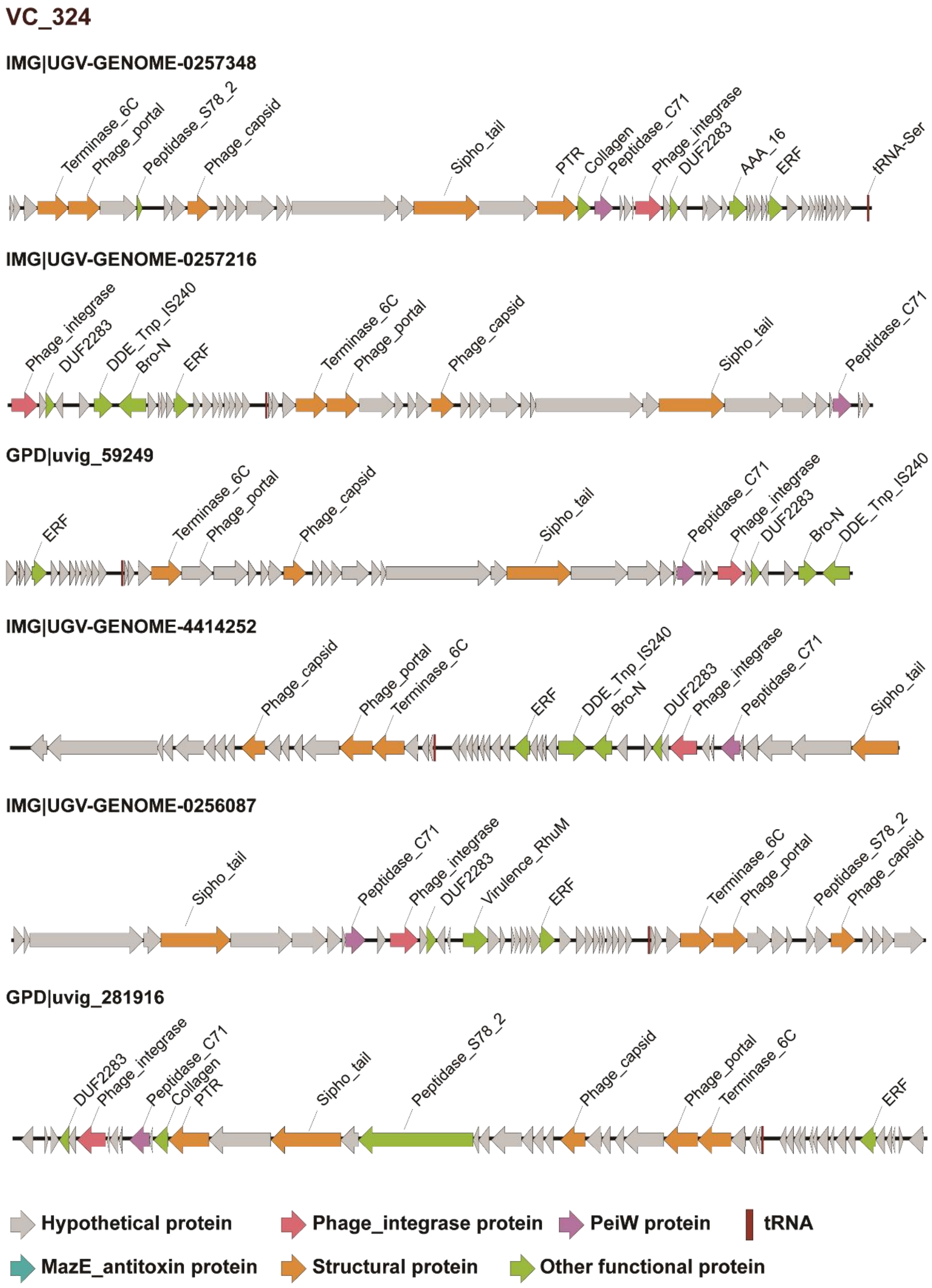
VC_324
IMG|UGV-GENOME-0257348
Terminase_6C
Phage_portal
Peptidase_S78_2
Phage_capsid
Sipho_tail
PTR
Collagen
Peptidase_C71
Phage_integrase
DUF2283
AAA_16
ERF
tRNA-Ser
IMG|UGV-GENOME-0257216
Phage_integrase
DUF2283
DDE_Tnp_IS240
Bro-N
ERF
Terminase_6C
Phage_portal
Phage_capsid
Sipho_tail
Peptidase_C71
GPD|uvig_59249
ERF
Terminase_6C
Phage_portal
Phage_capsid
Sipho_tail
Peptidase_C71
Phage_integrase
DUF2283
Bro-N
DDE_Tnp_IS240
IMG|UGV-GENOME-4414252
Phage_capsid
Phage_portal
Terminase_6C
ERF
DDE_Tnp_IS240
Bro-N
DUF2283
Phage_integrase
Peptidase_C71
Sipho_tail
IMG|UGV-GENOME-0256087
Sipho_tail
Peptidase_C71
Phage_integrase
DUF2283
Virulence_RhuM
ERF
Terminase_6C
Phage_portal
Peptidase_S78_2
Phage_capsid
GPD|uvig_281916
DUF2283
Phage_integrase
Peptidase_C71
Collagen
PTR
Sipho_tail
Peptidase_S78_2
Phage_capsid
Phage_portal
Terminase_6C
ERF
Hypothetical protein
Phage_integrase protein
PeiW protein
tRNA
MazE_antitoxin protein
Structural protein
Other functional protein

VC_324

provir|Zeller_G_2014_MSB_CCIS95409808ST-4-0.NODE_65_149891_1

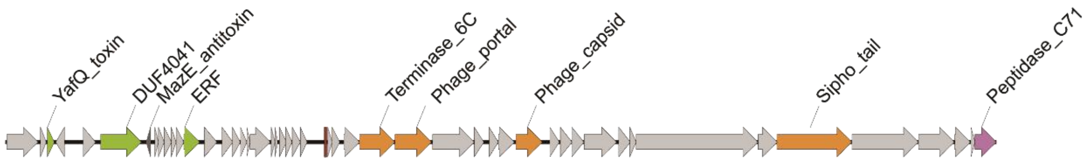

provir|Zeller_G_2014_MSB_CCIS90164298ST-4-0.NODE_164_78593_1

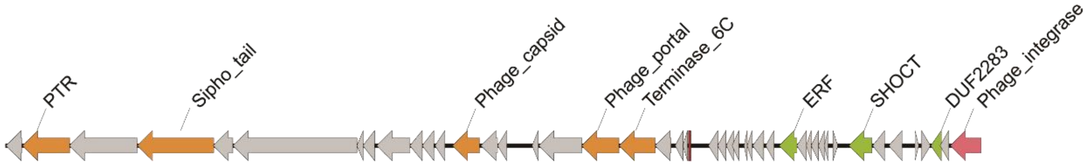

provir|Rampelli_S_2020_mSystems_SRR9654999.NODE_52_92916_1

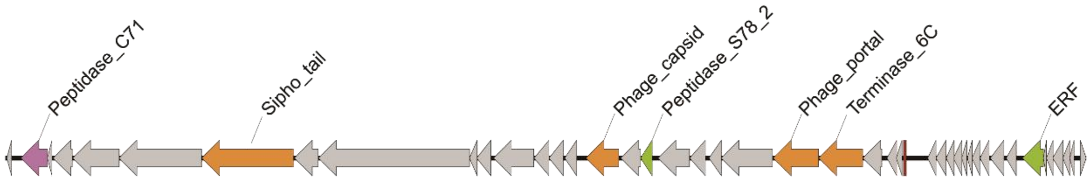

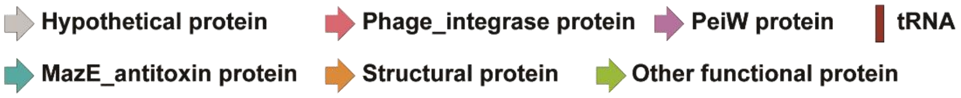

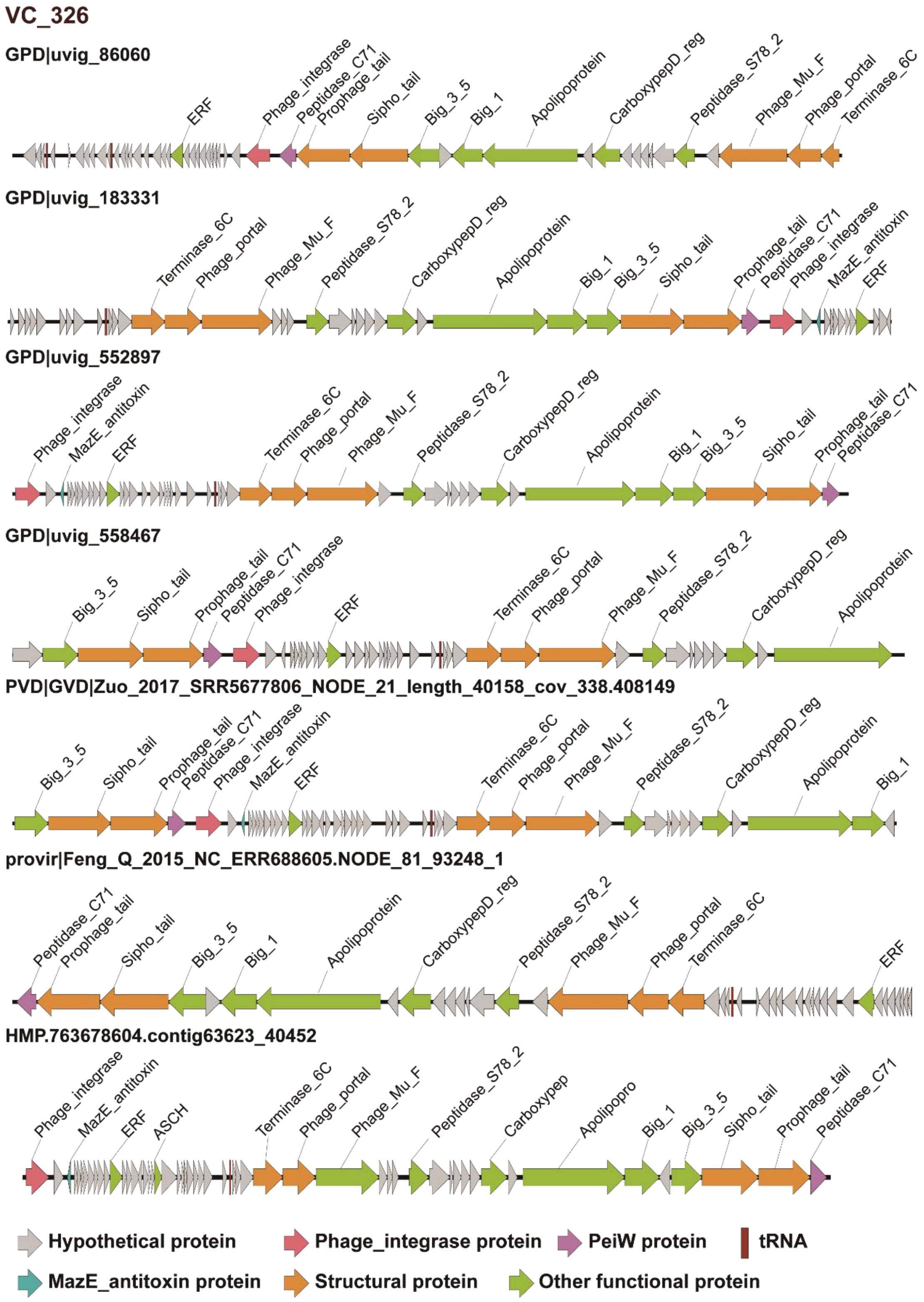
VC_326
GPD|uvig_86060
ERF
Phage_integrase
Peptidase_C71
Prophage_tail
Sipho_tail
Big_3_5
Big_1
Apolipoprotein
CarboxypepD_reg
Peptidase_S78_2
Phage_Mu_F
Phage_portal
Terminase_6C
GPD|uvig_183331
Terminase_6C
Phage_portal
Phage_Mu_F
Peptidase_S78_2
CarboxypepD_reg
Apolipoprotein
Big_1
Big_3_5
Sipho_tail
Prophage_tail
Peptidase_C71
Phage_integrase
MazE_antitoxin
ERF
GPD|uvig_552897
Phage_integrase
MazE_antitoxin
ERF
Terminase_6C
Phage_portal
Phage_Mu_F
Peptidase_S78_2
CarboxypepD_reg
Apolipoprotein
Big_1
Big_3_5
Sipho_tail
Prophage_tail
Peptidase_C71
GPD|uvig_558467
Big_3_5
Sipho_tail
Prophage_tail
Peptidase_C71
Phage_integrase
ERF
Terminase_6C
Phage_portal
Phage_Mu_F
Peptidase_S78_2
CarboxypepD_reg
Apolipoprotein
PVD|GVD|Zuo_2017_SRR5677806_NODE_21_length_40158_cov_338.408149
Big_3_5
Sipho_tail
Prophage_tail
Peptidase_C71
Phage_integrase
MazE_antitoxin
ERF
Terminase_6C
Phage_portal
Phage_Mu_F
Peptidase_S78_2
CarboxypepD_reg
Apolipoprotein
Big_1
provir|Feng_Q_2015_NC_ERR688605.NODE_81_93248_1
Peptidase_C71
Prophage_tail
Sipho_tail
Big_3_5
Big_1
Apolipoprotein
CarboxypepD_reg
Peptidase_S78_2
Phage_Mu_F
Phage_portal
Terminase_6C
ERF
HMP.763678604.contig63623_40452
Phage_integrase
MazE_antitoxin
ERF
ASCH
Terminase_6C
Phage_portal
Phage_Mu_F
Peptidase_S78_2
Carboxypep
Apolipopro
Big_1
Big_3_5
Sipho_tail
Prophage_tail
Peptidase_C71
Hypothetical protein
Phage_integrase protein
PeiW protein
tRNA
MazE_antitoxin protein
Structural protein
Other functional protein

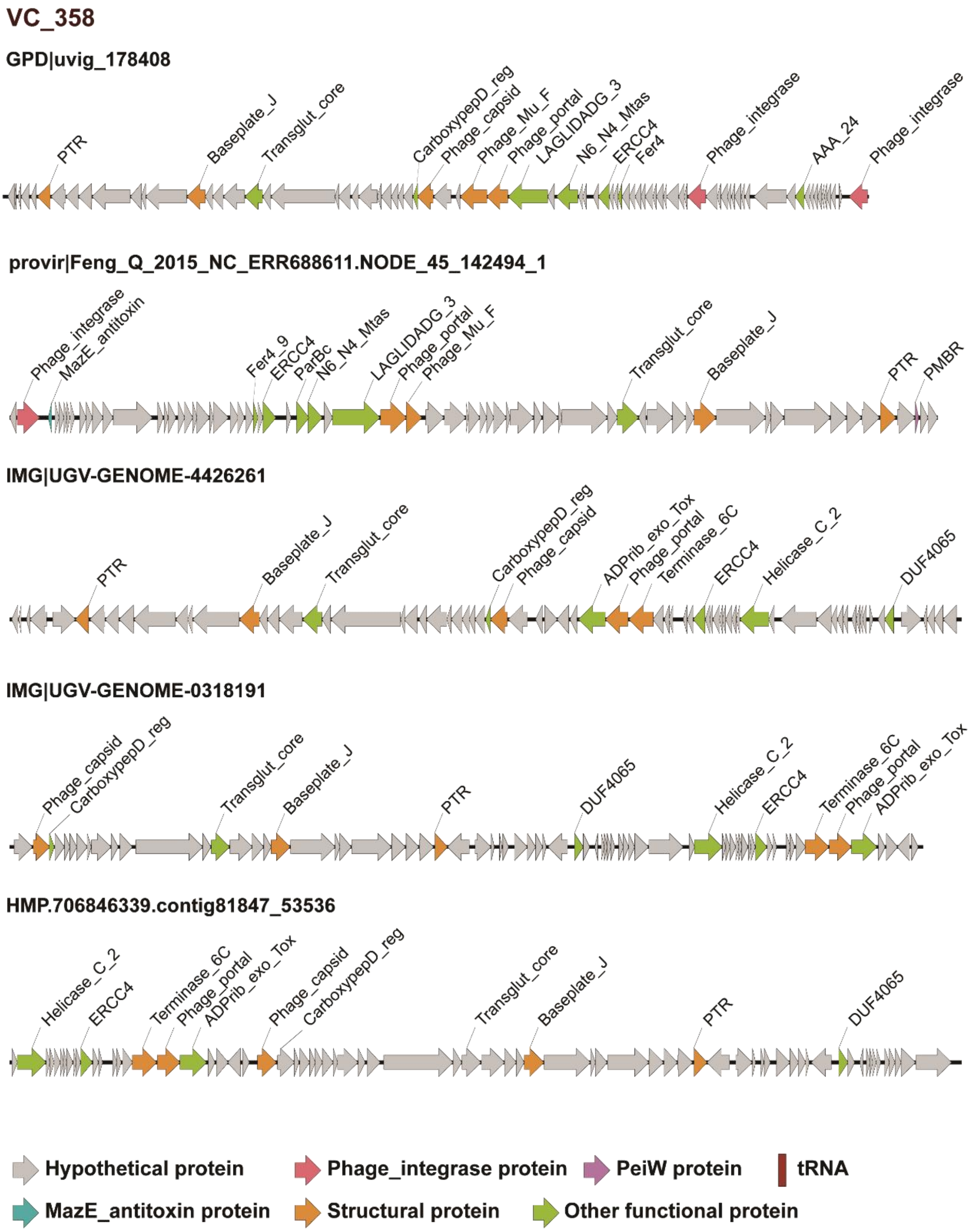
VC_358
GPD|uvig_178408
PTR
Baseplate_J
Transglut_core
CarboxypepD_reg
Phage_capsid
Phage_Mu_F
Phage_portal
LAGLIDADG_3
N6_N4_Mtas
ERCC4
Fer4
Phage_integrase
AAA_24
Phage_integrase
provir|Feng_Q_2015_NC_ERR688611.NODE_45_142494_1
Phage_integrase
MazE_antitoxin
Fer4_9
ERCC4
ParBc
N6_N4_Mtas
LAGLIDADG_3
Phage_portal
Phage_Mu_F
Transglut_core
Baseplate_J
PTR
PMBR
IMG|UGV-GENOME-4426261
PTR
Baseplate_J
Transglut_core
CarboxypepD_reg
Phage_capsid
ADPrib_exo_Tox
Phage_portal
Terminase_6C
ERCC4
Helicase_C_2
DUF4065
IMG|UGV-GENOME-0318191
Phage_capsid
CarboxypepD_reg
Transglut_core
Baseplate_J
PTR
DUF4065
Helicase_C_2
ERCC4
Terminase_6C
Phage_portal
ADPrib_exo_Tox
HMP.706846339.contig81847_53536
Helicase_C_2
ERCC4
Terminase_6C
Phage_portal
ADPrib_exo_Tox
Phage_capsid
CarboxypepD_reg
Transglut_core
Baseplate_J
PTR
DUF4065
Hypothetical protein
Phage_integrase protein
PeiW protein
tRNA
MazE_antitoxin protein
Structural protein
Other functional protein

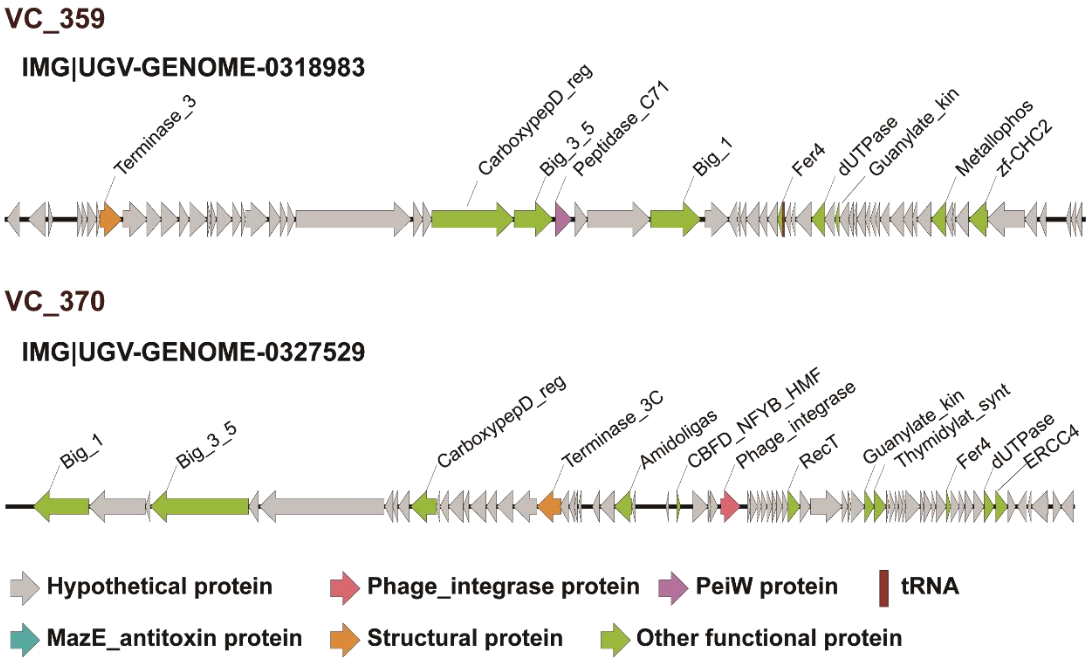

Supplementary Fig. 13 Genetic maps of the 33 complete *Caudoviricetes* genomes in HGAVD. The arrows depict the location and direction of predicted proteins on the viral genomes, and the filled colors indicate different gene functional categories, as depicted in the legend. The annotations were based on searches against the Pfam database, and only significant results (e-value < 1e–5) are shown.

References

1. Parks, D.H. et al. A complete domain-to-species taxonomy for Bacteria and Archaea. *Nat Biotechnol* **38**, 1079-1086 (2020).
2. Almeida, A. et al. A unified catalog of 204,938 reference genomes from the human gut microbiome. *Nat Biotechnol* **39**, 105-114 (2021).
3. Chibani, C.M. et al. A catalogue of 1,167 genomes from the human gut archaeome. *Nat Microbiol* **7**, 48-61 (2022).
4. Hunter, J.D. Matplotlib: a 2D graphics environment. *Comput Sci Eng* **9**, 90–95 (2007).
