# Supplementary material for: Metagenomic analysis reveals unexplored diversity of archaeal virome in the human gut
Source: Nat Commun. 2022 Dec 29;13:7978. doi: 10.1038/s41467-022-35735-y (PMC9800368; doi:10.1038/s41467-022-35735-y)
Supplement: Supplementary file 3 — Description of Additional Supplementary Files [file 41467_2022_35735_MOESM3_ESM.docx]

**Supplementary Data legends**

**Supplementary Data 1.** Collection of metagenomic datasets in this study, Related to Supplementary Figure 1.

**Supplementary Data 2.** Taxonomic classification of 2,948 Archaeal contigs.

**Supplementary Data 3.** **a**. 1,162 gut archaeal genomes collected from UHGG (Unified Human Gastrointestinal Genome). **b**. 833 gut archaeal genomes in UHGG were matched to the 15,732 archaeal contigs (≥ 3kbp) identified from metagenomic samples.

**Supplementary Data 4**. **a**. Metadata of 1,904 individuals. **b**. Relative abundance table for 56 gut archaeal species in each sample. **c**. Relative abundance table for 22 gut archaeal genera in each sample.

**Supplementary Data 5.** The number of Spacers derived from archaeal contigs and archaeal genomes.

**Supplementary Data 6.** Metadata for 1,279 representative contigs in HGAVD. Fields include:

1. Viral Contig ID: representative archaeal viral contig identifier
2. Spacer Sources: the spacers matched to the viral contigs
3. Length: contig length in bp, if the contigs were predicted as provirus by CheckV, the sequence length of the viral region is displayed on the column of Provirus_length
4. Viral Taxonomy: Predicted viral taxon of the contig
5. Taxonomic classification method: the method used for viral taxonomic classification
6. Host_Phylum, Host_Class, Host_Order, Host_Family, Host_Genus, Host_Species: Taxonomic classification of viral host
7. Viral_Cluster: Viral cluster to which the contig belongs
8. Checkv_provirus: whether or not the contig was flanked by DNA from the host (these regions were removed)
9. Provirus_length: the sequence length of the viral region
10. checkv_quality: medium quality (50-90% complete), high quality (>90% complete), complete (closed genome)
11. Checkv_completeness: CheckV estimated completeness
12. Checkv_contamination: CheckV estimated contamination
13. Source Location: country of origin of Metagenomic_runs_detected
14. Prevalence: the percentage of individuals with the archaeal viruses in their gut
15. VirSorter category: The category for each of the input sequences predicted by Virsoter.
16. Virsorter2: The result for each of the input sequences predicted by Virsoter2.
17. VirFinder score: The score for each of the input sequences predicted by VirFinder.
18. VirFinder p-value: The p-value for each of the input sequences predicted by VirFinder.
19. DeepVirFinder score: The score for each of the input sequences predicted by DeepVirFinder.
20. DeepVirFinder p-value: The p-value for each of the input sequences predicted by DeepVirFinder.

**Supplementary Data 7.** Network analysis for viral taxonomic classification.

**Supplementary Data 8.** Network analysis for comparison with public viral databases.

**Supplementary Data 9.** Relative abundance table for 1279 gut archaeal viral species in each sample.

**Supplementary Data 10.** ANOSIM analysis for archaeal viruses.

**Supplementary Data 11.** Genomic context for archaeal virus IMG|UGV-GENOME-0271153 and virus IMG|UGV-GENOME-0263128.

**Supplementary Data 12.** Distribution of genes on the VCs.

**Supplementary Data 13.** Genomic context for complete 36 archaeal viral genomes in HGAVD.

**Supplementary Data 14.** The sample list for estimating the fraction of archaeal viruses in human gut virome.

**Supplementary Data 15.** Taxonomic information of 33 archaeal contigs identified from the oral, skin, and vagina samples.
