# Supplementary material for: Metagenomic analysis reveals unexplored diversity of archaeal virome in the human gut
Source: Nat Commun. 2022 Dec 29;13:7978. doi: 10.1038/s41467-022-35735-y (PMC9800368; doi:10.1038/s41467-022-35735-y)
Supplement: Supplementary file 20 — Reporting Summary [file 41467_2022_35735_MOESM20_ESM.pdf]

nature portfolio

Corresponding author(s): Ma, Yingfei

Last updated by author(s): Dec 12, 2022

Reporting Summary

Nature Portfolio wishes to improve the reproducibility of the work that we publish. This form provides structure for consistency and transparency in reporting. For further information on Nature Portfolio policies, see our Editorial Policies and the Editorial Policy Checklist.

Statistics

For all statistical analyses, confirm that the following items are present in the figure legend, table legend, main text, or Methods section.

n/a	Confirmed	
☐	☒	The exact sample size (*n*) for each experimental group/condition, given as a discrete number and unit of measurement
☒	☐	A statement on whether measurements were taken from distinct samples or whether the same sample was measured repeatedly
☐	☒	The statistical test(s) used AND whether they are one- or two-sided<br>*Only common tests should be described solely by name; describe more complex techniques in the Methods section.*
☐	☒	A description of all covariates tested
☐	☒	A description of any assumptions or corrections, such as tests of normality and adjustment for multiple comparisons
☐	☒	A full description of the statistical parameters including central tendency (e.g. means) or other basic estimates (e.g. regression coefficient) AND variation (e.g. standard deviation) or associated estimates of uncertainty (e.g. confidence intervals)
☐	☒	For null hypothesis testing, the test statistic (e.g. *F*, *t*, *r*) with confidence intervals, effect sizes, degrees of freedom and *P* value noted<br>*Give P values as exact values whenever suitable.*
☐	☒	For Bayesian analysis, information on the choice of priors and Markov chain Monte Carlo settings
☒	☐	For hierarchical and complex designs, identification of the appropriate level for tests and full reporting of outcomes
☒	☐	Estimates of effect sizes (e.g. Cohen's *d*, Pearson's *r*), indicating how they were calculated

Our web collection on statistics for biologists contains articles on many of the points above.

Software and code

Policy information about availability of computer code

Data collection	National Center for Biotechnology and Information (NCBI), IMG/VR database v3, Gut Phage Database (GPD), GTDB database release 95, the Earth's Virome database (EVP), Human Gut Virome database (HGV), Uncultured Viral Database of Archaeal and Bacteria (GL-UVAB), Pfam. v32 database
Data analysis	The following software was used: CheckM v1.0.11; Bowtie v2.3.2; HMMER v3.1b2; IQ-TREE v1.5.5; iTol v6; VirSorter v1.0.3; NCBI blast+ v2.9; SPAdes v3.10.0; Prodigal v. 2.6.3; CD-HIT v4.6; GTDB-Tk v0.3.3; GTDB-Tk v0.3.1; CheckV v0.6.0; VirFinder v1.1; DeepVirFinder v1.0; vConTACT v2.0; eggNOG-mapper v2.0.0; R version 4.0.5; MEGA X; Soap2 v2.21; RAxML v8; CRISPR Recognition Tool v1.1; VirSorter2 v2.2.3; IQ-TREE v1.6.12;

For manuscripts utilizing custom algorithms or software that are central to the research but not yet described in published literature, software must be made available to editors and reviewers. We strongly encourage code deposition in a community repository (e.g. GitHub). See the Nature Portfolio guidelines for submitting code & software for further information.

Data

Policy information about availability of data

All manuscripts must include a data availability statement. This statement should provide the following information, where applicable:

- Accession codes, unique identifiers, or web links for publicly available datasets
- A description of any restrictions on data availability
- For clinical datasets or third party data, please ensure that the statement adheres to our policy

The annotated nucleotide sequences of archaeal viruses (FASTA + GFF), archaeal viral hallmark genes, accompanied with the metadata file describing the origin of each contig, taxonomy, including VC, host prediction information, completeness score are available in the link https://doi.org/10.6084/m9.figshare.21152404.v3.

Human research participants

Policy information about studies involving human research participants and Sex and Gender in Research.

Reporting on sex and gender	*Use the terms sex (biological attribute) and gender (shaped by social and cultural circumstances) carefully in order to avoid confusing both terms. Indicate if findings apply to only one sex or gender; describe whether sex and gender were considered in study design whether sex and/or gender was determined based on self-reporting or assigned and methods used. Provide in the source data disaggregated sex and gender data where this information has been collected, and consent has been obtained for sharing of individual-level data; provide overall numbers in this Reporting Summary. Please state if this information has not been collected. Report sex- and gender-based analyses where performed, justify reasons for lack of sex- and gender-based analysis.*
Population characteristics	*Describe the covariate-relevant population characteristics of the human research participants (e.g. age, genotypic information, past and current diagnosis and treatment categories). If you filled out the behavioural & social sciences study design questions and have nothing to add here, write "See above."*
Recruitment	*Describe how participants were recruited. Outline any potential self-selection bias or other biases that may be present and how these are likely to impact results.*
Ethics oversight	*Identify the organization(s) that approved the study protocol.*

Note that full information on the approval of the study protocol must also be provided in the manuscript.

Field-specific reporting

Please select the one below that is the best fit for your research. If you are not sure, read the appropriate sections before making your selection.

☐ Life sciences ☐ Behavioural & social sciences ☒ Ecological, evolutionary & environmental sciences

For a reference copy of the document with all sections, see nature.com/documents/nr-reporting-summary-flat.pdf

Ecological, evolutionary & environmental sciences study design

All studies must disclose on these points even when the disclosure is negative.

Study description	We conducted a comprehensive analysis of the archaeal viruses from the human gut metagenomes and the existing virus collections using the CRISPR spacer and viral signature-based approach. This resulted in 1,279 viral species, of which, 95.2% infected Methanobrevibacteria_A, 56.5% shared high identity (> 95%) with the archaeal proviruses, 37.2% had a host range across archaeal species, and 55.7% were highly prevalent in the human population (>1%).
Research sample	The aim was to include publicly available human microbial metagenomic datasets (n = 3,971) from 12 studies and encompass 1,904 individuals across rural and urban populations representing 13 countries from 4 continents. In the meantime, the number of samples from each countries was generally more than 50 to ensure geographically diverse.
Sampling strategy	Sampling strategy was not relevant to this study, as we analysed publicly available data.
Data collection	Data collection is described in "Collection of metagenomic sequencing data sets used for this study"
Timing and spatial scale	This study exclusively included a total of 3,971 publicly available human gut metagenome samples representing 13 countries from 4 continents. Timing scale was not applicable as we analyzed publicly available data without experiments.
Data exclusions	No data were excluded from the analyses.
Reproducibility	All analyses can be reproduced based on the datasets and information provided. The manuscript contains no wet-lab experiments.

Randomization	Randomization was not relevant to this study, as we analysed publicly available data.
Blinding	Not relevant to this study, as we analysed publicly available data.

Did the study involve field work? ☐ Yes ☒ No

Reporting for specific materials, systems and methods

We require information from authors about some types of materials, experimental systems and methods used in many studies. Here, indicate whether each material, system or method listed is relevant to your study. If you are not sure if a list item applies to your research, read the appropriate section before selecting a response.

Materials & experimental systems

n/a	Involved in the study
☒	☐ Antibodies
☒	☐ Eukaryotic cell lines
☒	☐ Palaeontology and archaeology
☒	☐ Animals and other organisms
☒	☐ Clinical data
☒	☐ Dual use research of concern

Methods

n/a	Involved in the study
☒	☐ ChIP-seq
☒	☐ Flow cytometry
☒	☐ MRI-based neuroimaging
